# Leveraging Wikipedia in undergraduate health sciences education: a key tool for information literacy and knowledge translation

**DOI:** 10.29173/jchla29688

**Published:** 2023-12-01

**Authors:** Denise Smith

**Affiliations:** Librarian III, Health Sciences Library, McMaster University, Hamilton, ON

## Abstract

**Background:**

Academic institutions and libraries are familiar with Wikipedia. There is growing momentum in higher education for using Wikipedia as a learning tool in various contexts. These include, but are not limited to, the use of Wikipedia-based assignments to teach information literacy, science communication, evidence-based practice, and more. Although there is growing acceptance of Wikipedia’s value in the classroom, there are limited exemplars available for how it is applied in undergraduate health sciences education.

**Description:**

This program description describes a librarian instructed course in the Bachelor of Health Sciences Program at McMaster University in which students dedicate one academic term to learning about Wikipedia content production and making contributions to a health-related Wikipedia article of their choice.

**Outcomes:**

In the five iterations of this course that have been offered, undergraduate health sciences students have made significant contributions to 25 health-related articles in Wikipedia. They have added more than 120,000 words and over 2,000 references to high-quality literature. In class, conversations emerged about the meaningfulness of the editing Wikipedia, information literacy, and knowledge translation.

## Introduction

Academic institutions and libraries are not strangers to Wikipedia. Historically, the resource has been stigmatized in academia [1], but some educators have embraced Wikipedia for its broad reach and its utility as an educational tool [2–4]. The peer-reviewed literature offers a breadth of rationales, foci, and exemplars for the inclusion of Wikipedia editing in a course, in an assignment, or in library instruction sessions. These include, but are not limited to, decolonizing information and educational curricula [5], understanding core concepts in a variety of disciplines ranging from STEM to the humanities [6] , teaching evidence based practice in health fields [7– 11], and teaching information literacy [12,13]. However, there is little coverage of Wikipedia-based assignments in undergraduate health education.

In the context of academic librarianship, the inclusion of Wikipedia-based work is easily mapped onto the Association for College and Research Libraries (ACRL) *Framework for Information Literacy for Higher Education* [14]. Namely, the construction and contextuality of authority, the process of information creation, and the value of information. Further, through the lens of critical information literacy [15,16], the inclusion of Wikipedia-based work in higher education has also been argued to improve knowledge equity alongside the development of students’ understanding of systemic biases [17,18]. Such bias, which Wikipedia has long been known to mirror [19,20], was thrust into the spotlight in 2021 when it was discovered that Canadian Nobel Prize winner, Donna Strickland, did not have a Wikipedia page on the grounds that she did not meet Wikipedia’s notability requirements [21]. Despite gaining traction as a valuable learning tool in higher education, the prevalence of Wikipedia use in undergraduate health sciences education continues to be limited.

Undergraduate students are in a unique position. They are not yet expected to produce new knowledge, as expected at the graduate level. However, it is commonly anticipated that upper year undergraduate students have mastered rudimentary information literacy skills. They are often expected to understand what information they require to answer a research question they have posed, how to search and retrieve relevant information, and how to appraise the information they retrieve. These students also have access: as tuition paying members of an academic institution, students are in the advantageous position of not having to contend with paywalls. They have nearly unfettered access to thousands of clinical and academic peer-reviewed publications. This combination of skills and access positions upper year undergraduate students as an ideal population for making contributions to Wikipedia.

## Description

The Bachelor of Health Sciences (BHSc) program at McMaster University is an inquiry-based, interdisciplinary four-year undergraduate program. More than 60% of its graduates have gone on to attend medical school and with a focus on keeping class sizes small, entry into the program is limited to approximately 260 students. The BHSc program employs inquiry-based learning [22], which places the instructor, called a facilitator, in the role of guide as students direct their own learning. Each year, students select an Inquiry course section to participate in. Each Inquiry section is individual in both topic and structure. Some focus on health-specific topics, while others are more conceptual. Some Inquiry course sections have a facilitator who offers guidance and support but the course is otherwise unstructured with students constructing and directing their own learning path. Other Inquiry course sections offer more structure but still situate the students at the centre of their own learning who can chart and pursue their own path towards their learning objectives.

Employing the Inquiry model described above, this course offers students a moderately structured environment to explore their own learning. To set the stage for Wikipedia authorship, the first three weeks of the course are dedicated to reflecting on information production and questioning the dominant paradigm of what constitutes authoritative information. Students are invited to reflect on the contextuality of authority and explore the myriad variables that influence the production of health information resources that are commonly believed to be high-quality and evidence-based, such as Canada’s Food Guide and high-impact clinical journals. At the same time that students are thinking outside the evidence-based medicine box, they are assigned training modules through WikiEducation, a program that provides resources to facilitate or support Wikipedia editing assignments or courses. Students simultaneously learn how to edit Wikipedia’s health and medical information, including guidelines and procedures, while gaining an alternative perspective on the value of health information. Given the rigorous contribution policies and guidelines for authoring health and medical articles on Wikipedia, the training is the most structured component of the course.

Early in the course, students are encouraged to form small groups and select a health-related topic and relevant Wikipedia article. The selected Wikipedia article must require improvement in some capacity. This can be extensive review and overhaul and adding up-to-date citations to a moderately developed article. It could also be a Stub or Start class article that requires significant expansion. Each group submits a project plan that outlines their topic and article of choice, a rationale for their choice, an analysis of the article in its current state, and a list of S.M.A.R.T goals [23] for their group contributions over the course of the term. Following the WikiEducation program [24], the WikiEdu Dashboard is used to identify milestones - such as completion of training modules provided by WikiEducation, creating an account, and adding a citation to an existing article - and to monitor student progress.

Approximately three to four weeks into their editing journey, each group submits a project progress report. This update is an opportunity for students to reassess their initial project plan, the feasibility of their goals, and adjust their path moving forward, if required. The project progress report is crucial. Students are infrequently provided an opportunity to shift gears once they have started a project, but this does not necessarily reflect the reality of everyday life. The progress report allows students to reflect on their progress so far and, with the added perspective of having already begun to make live edits to an article, can rethink their initial, often ambitious, goals. Furthermore, this progress report incentivizes students to begin editing a live Wikipedia article as early as possible in the term. In the five iterations of this course, students were consistent in their hesitation to begin editing live Wikipedia articles. However, once the students gained confidence after their first few contributions, they would rapidly gain momentum.

At the end of the term, each group of students presents a project report to share their contributions to Wikipedia and the impact of those contributions. Students are invited to share quantitative measures of their impact, such as pageviews since they began editing, but are also encouraged to reflect on the meaningfulness of their contributions (e.g., what does it mean to have contributed to a widely read information resource?). This report is presented in-class for the benefit of all students.

## Outcomes

Since 2019, this course has been offered five times. Cumulatively, a total of 64 student editors have made contributions to 25 health-related articles in Wikipedia (Table 1. Student Contributions to Wikipedia). At the end of this course students are able to:

- critically analyze traditionally authoritative sources of health information and critically engage with non-traditional sources;
- contribute to the health information landscape as participatory producers of high-quality health information for public dissemination;
- understand that the health information landscape is complex, and that students have a role within it;
- identify strengths and challenges of health information in its different formats.

**Table 1:** Student Contributions to Wikipedia

Iteration	Student Editors	Articles Edited	Words Added(thousands)	References Added
Winter 2019	9	6	12.2	163
Fall 2019	19	7	53.3	620
Fall 2020	20	7	36.5	610
Fall 2021	11	4	41.4	577
Fall 2022	5	1	13.871	158
**Total**	**64**	**25**	**120.771**	**2,128**

Furthermore, some students who enrolled in this course went on to continue contributing to Wikipedia as an independent project or for credit for their fourth-year project. This outcome speaks to the ignited spark that some students reported when asked to reflect on the course and their contributions to Wikipedia.

Students were not formally interviewed, however some broad themes emerged in class discussions that might help situate the students’ perceptions of their own learning. At the end of the term students reflected on the meaningfulness of contributing to Wikipedia compared to writing a term paper. There were also conversations about their strengthened ability to scrutinize the information they find online, in Wikipedia and beyond. Many students continued to demonstrate a healthy skepticism about the quality of information on Wikipedia and online in general, but also expressed a new confidence in their ability to identify when the information in Wikipedia might be suitable for their needs or the needs of others, and when different sources might be more appropriate. Conversation about knowledge translation also emerged. Namely, the translation of complex health and medical language into plain, easy to understand language. Finally, some students went on to continue to make improvements to Wikipedia both informally, on their own time, and formally, as part of a more intensive fourth-year project.

## Discussion

Wikipedia authorship for course credit is not a ground-breaking innovation in higher education . There are numerous examples of individual assignments or entire courses that require students to make contributions to Wikipedia[25]. Although there are examples of undergraduate students editing Wikipedia in topics outside of health and medicine, there is a focus in the peer-reviewed literature on health professions students, such as students of medicine, pharmacy, occupational therapy, or dentistry. This program description provides insight into the value and utility of undergraduate students in the health sciences as skilled and discerning contributors to Wikipedia’s health and medical content. It also provides insight into the value, potential, and utility of a Wikipedia editing assignment as an educational tool for undergraduate students in the health sciences.

As students of health at McMaster University, evidence-based practice plays an influential role in students’ perceptions of what constitutes authoritative and credible information. While valuable, the critical appraisal skills that an evidence-based practice framework teaches students contributes to a preference for health evidence that the public does not necessarily have access to. As future professionals who will likely engage with members of the public in some capacity, teaching undergraduate health sciences students to edit Wikipedia has a two-fold impact. First, students are presented with an opportunity to learn a hard skill (how to edit Wikipedia) and strengthen soft skills (information appraisal, knowledge translation). Second, students learn that in some contexts, and for many members of the public, Wikipedia is a valuable and credible health information resource and that the “best evidence” is largely inaccessible to much of the public both literally in the form of paywalls, and more figuratively, in that traditionally preferred sources of health evidence (such as peer-reviewed studies published in academic or clinical journals) use profession-specific language that is not easily understood by non-experts.

In the course, students evolve from a state of information consumer to the role of information producers. They produce openly available content that contributes to knowledge sharing. Students also engage in collaborative production with their peers in-class and with Wikipedia editors with whom engagement is required to seek guidance, respond to feedback, or even resolve disputes. Dispute resolution was encountered by a minority of student contributors. In these situations, students may have had their contributions reverted for a variety of reasons: the source cited did not align with Wikipedia’s reliability guidelines for medical information sources; the content added was of questionable value to the article; or student editors violated some other editing policy (e.g. Neutral Point of View, No Original Research) as they adjusted to editing Wikipedia. In these situations, the course instructor supports or mediates discussion between the students and other editors. The majority of these experiences have been positive. The Wikipedia community is generally supportive and encouraging of student contributors.

Librarians and libraries are in a unique and advantageous position. This course, facilitated and designed by a librarian, demonstrates that librarians have the expertise and value of engaging students in the classroom to apply information literacy in a real-world setting that has real time outcomes. Whether in a formal for-credit academic course, or as part of a library’s suite of instructional sessions, libraries are ideal facilitators for student authorship in Wikipedia. Library workers are familiar with the resources that students have access to and they are typically the university’s leading experts in information literacy.

Wikipedia is widely regarded as a dubious tertiary resource. Leveraging the skills of upper year undergraduate students, and their access to a wealth of materials, is of mutual benefit. Students gain pragmatic and transferable skills in information assessment, knowledge translation, and information literacy. Wikipedia benefits from improvements to its content, consequently benefiting the potentially millions of individuals that consume student contributions within the article.
